# Current Progress and Perspectives on Using Gold Compounds for the Modulation of Tumor Cell Metabolism

**DOI:** 10.3389/fchem.2021.733463

**Published:** 2021-08-09

**Authors:** Leiya Kou, Shuang Wei, Pei Kou

**Affiliations:** ^1^ The First Clinical College, Hubei University of Chinese Medicine, Wuhan, China; ^2^ Department of Respiratory and Critical Care Medicine, Key Laboratory of Pulmonary Diseases of Health Ministry, Tongji Hospital, Tongji Medical College Huazhong University of Science and Technology, Wuhan, China; ^3^ Department of Medical Record, Wuhan No. 1 Hospital, Wuhan, China

**Keywords:** anticancer therapy, gold compounds, tumor cell metabolism, glucose metabolism, protein metabolism, nucleic acid metabolism

## Abstract

Altered cellular metabolism, which is essential for the growth and survival of tumor cells in a specific microenvironment, is one of the hallmarks of cancer. Among the most significant changes in the metabolic pattern of tumor cells is the shift from oxidative phosphorylation to aerobic glycolysis for glucose utilization. Tumor cells also exhibit changes in patterns of protein and nucleic acid metabolism. Recently, gold compounds have been shown to target several metabolic pathways and a number of metabolites in tumor cells. In this review, we summarize how gold compounds modulate glucose, protein, and nucleic acid metabolism in tumor cells, resulting in anti-tumor effects. We also discuss the rationale underlying the anti-tumor effects of these gold compounds and highlight how to effectively utilize against various types of tumors.

## Introduction

All cells need energy to survive. In particular, tumor cells have a high energy requirement due to their high proliferation rates. Changes in the metabolic patterns of tumor cells not only provide materials for cell growth, but also provide signals for continuous cell proliferation to allow the survival and growth of tumor cells in specific microenvironments. The tumor cells’ ability to change cellular metabolic patterns, also known as metabolic reprogramming, is characterized by a dysregulated glucose uptake that is, markedly different from that exhibited by normal cells (Pavlova and Thompson, 2016). This phenomenon was first observed by German Dr. Otto Warburg in 1926, wherein he found that some cells absorbed nutrients differently from others and that normal cells use oxygen to convert food into energy, while cancer cells prefer glycolysis for energy production (“Warburg effect”) (Warburg et al., 1927).

Recent advances in the study of glucose uptake and metabolism in tumor cells have enabled us to understand how metabolic reprogramming allows tumor cell growth and proliferation. Lactic acid produced through glycolysis provides fuel for tumor growth and regulates the tumor microenvironment (TME), making the TME conducive to angiogenesis and tumor invasion (Bonuccelli et al., 2010). The discovery of glycolysis has laid a foundation for the field of tumor metabolism, and, subsequently, an increasing number of scientists have focused on the field of tumor metabolism. In addition to glucose metabolism, protein synthesis, and catabolism are also altered in tumor tissues, mainly manifesting as the enhanced breakdown of proteins into amino acids for cyclical utilization in protein synthesis. Protein metabolism in tumor cells may involve enzymes such as proteasomes, which are considered potential targets of anti-tumor treatment using gold compounds (Milacic et al., 2006). Gold compounds can also regulate nucleic acid anabolism in tumor cells and exert anti-tumor effects through this mechanism (Galassi et al., 2020).

In 1965, Rosenberg discovered that electrolytic products from a platinum electrode inhibited *Escherichia coli* cell division (Rosenberg et al., 1965), and, in 1969, he discovered that platinum compounds had anticancer activity (Rosenberg et al., 1969). Subsequently, scientists have studied the medical value of platinum compounds. Platinum compounds have saved the lives of numerous cancer patients; however, side effects, and resistance due to platinum-based anticancer therapy have become major obstacles to the clinical success of these compounds (Dasari and Tchounwou, 2014). Thus, researchers have extended their studies to other metal compounds such as gold compounds. Gold compounds, such as auranofin (ARF), were initially used to treat rheumatoid arthritis, and were subsequently found to have inhibitory effects on various types of tumors; other gold (III) compounds can also be used in the treatment of platinum-resistant tumors (To et al., 2009; Hou et al., 2018). Here, we review the anti-tumor effects of gold compounds based on their effects on glucose, protein, and nucleic acid metabolism.

## The Effects of Gold Compounds on Glucose Metabolism in Tumors

Glucose is the primary energy source of cells and is an intermediate product of carbohydrate metabolism. Glucose metabolism in humans include aerobic and anaerobic glycolysis. Aerobic oxidation is the process by which glucose is oxidized to CO_2_ and H_2_O in the presence of oxygen (Nazaret et al., 2009). It is the main pathway for oxidative glucose metabolism and the main cellular pathway for energy production. Aerobic oxidation of glucose involves the following steps: glucose is first decomposed into pyruvate through glycolysis; then, pyruvate is oxidized and decarboxylated in the mitochondria to produce acetyl-CoA; this is then followed by the tricarboxylic acid cycle and oxidative phosphorylation (OXPHOS). Since Warburg’s discovery that glucose metabolism in tumor cells is mainly *via* aerobic glycolysis, an increasing number of researchers have focused on understanding role of glucose metabolism in the growth, proliferation, and metastasis of tumor cells. Tumor cells also metabolize glucose differently from normal cells in the OXPHOS pathway, and researchers have found that alterations in glucose metabolism contribute to the malignant proliferation of tumor cells (Solaini et al., 2011; Riganti et al., 2012).

Gold compounds have special electronic structures, variable reduction-oxidation (redox) states, and special biological activities. *In vitro* experiments have confirmed that some gold (I) and gold (III) compounds can affect glucose metabolism, mitochondrial function, and DNA synthesis in tumor cells, and tend to bind with proteins to produce anti-proliferation effects on tumors. Thus, gold (I) and gold (III) compounds have broad prospects for application as anti-tumor agents (Tiekink, 2008; Nobili et al., 2010).

### Gold Compounds and Glycolysis

**FIGURE 1:**
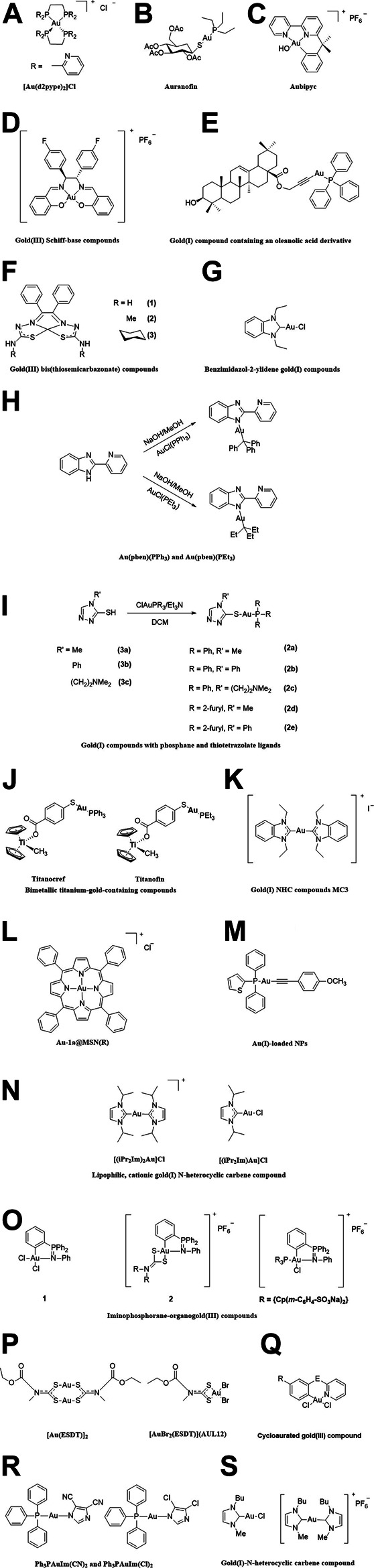
The chemical structure of the gold compounds mentioned in the paper. **(A)** [Au(d2pype)_2_]Cl; **(B)** Auranofin; **(C)** Aubipyc; **(D)** Gold(III) Schiff-base compounds; **(E)** Gold(I) compound containing an oleanolic acid derivative; **(F)** Gold(III) bis(thiosemicarbazonate) compounds; **(G)** Benzimidazol-2-ylidene gold(I) compounds; **(H)** Au(pben)(PPh_3_) and Au(pben)(PEt_3_); **(I)** Gold(I) compounds with phosphane and thiotetrazolate ligands; **(J)** Titanocref and Titanofin; **(K)** Gold(I) NHC compounds MC3; **(L)** Au-1a@MSN(R); **(M)** Au(I)-loaded NPs; **(N)** Lipophilic, cationic gold(I) N-heterocyclic carbene compound; **(O)** Iminophosphorane-organogold(III) compounds; **(P)** [Au(ESDT)]_2_ and [AuBr_2_(ESDT)] (AUL12); **(Q)** Cycloaurated gold(III) compound; **(R)** Ph_3_PAuIm(CN)_2_ and Ph_3_PAuIm(Cl)_2_; **(S)** Gold(I)-N-heterocyclic carbene compound.

One of the characteristics of tumor cells is the preference for glycolysis, which is characterized by significantly increased glucose uptake and lactic acid production even under normal oxygen conditions and normal mitochondrial function (Liberti and Locasale, 2016). Although glucose metabolism, such as glycolysis, is not common to all types of cancer, all tumor tissues exhibit increased glucose uptake. Pyruvate produced from glycolysis is metabolized into lactic acid by lactate dehydrogenase (LDH) to produce adenosine triphosphate (ATP) and provides energy for tumor growth (Gillies and Gatenby, 2007). We know that the amount of ATP produced through glycolysis is lower than that produced through OXPHOS, so why do rapidly growing tumor cells, which consume large amounts of glucose, use a metabolic mode with low productivity? Increasing evidence shows that changes in the metabolic patterns of cancer cells are largely associated with changes in the expression patterns of oncogenes and tumor suppressor genes, c-Myc, hypoxia inducible factor-1(HIF1), and p53 (Yeung et al., 2008; Levine and Puzio-Kuter, 2010). C-Myc is an oncogene, and c-Myc overexpression is associated with the incidence and recurrence of multiple human tumors (Hawksworth et al., 2010; Gabay et al., 2014). C-Myc can drive glycolysis; upregulate lactate dehydrogenase A (LDHA), hexokinase 2 (HK2), and pyruvate kinase M2 (PKM2); improve the transcription rate of glucose transporter (GLUT); regulate the transcription of monocarboxylate transporters (MCT); and promote tumor growth (Kim et al., 2007; Dang et al., 2009). In a study to investigate whether gold (I) compounds affect the proliferation of multiple myeloma (MM), human myeloma cells RPMI8226 were subcutaneously injected into 8-week-old female NOD/SCID mice and treated with a gold (I) phosphine compound ([Au (d2pype)_2_]Cl (Figure 1A), 5 mg/kg, intraperitoneal, Monday–Friday) for 2 weeks until the tumor size reached 30–50 mm^2^ (Sze et al., 2020). The results showed that [Au (d2pype)_2_]Cl significantly reduced c-Myc protein and mRNA levels in RPMI8226 tumors and effectively eradicated borteomi-resistant myeloma cells. The inhibitory effect of [Au (d2pype)_2_]Cl on RPMI8226 tumor cells may be related to GLUT, glycolytic enzymes, and metabolites regulated by c-Myc.

The phosphatidylinositol 3-kinase (PI3K)/Akt signaling pathway is related to proliferation, differentiation, and apoptosis, and is one of the most frequently altered pathways in several human tumors (Robbins and Hague, 2015; Chen et al., 2016; Yang et al., 2016). The PI3K/Akt pathway regulates the proliferation and survival of tumor cells and plays an important role in the migration, adhesion, and angiogenesis of tumor cells. The occurrence of tumors is related to excessive cell proliferation. Tumor proliferation requires a large amount of oxygen consumption; therefore, hypoxia is one of the basic characteristics of the solid TME. HIF1 is a transcription regulator widely found in mammalian and human cells, and it mediates the adaptation of tumor cells to hypoxic environments (Semenza, 2001). Studies have shown that the activation of PI3K/Akt in tumors can promote the expression of the downstream transcription factor HIF-1 even in the presence of sufficient oxygen, which, in turn, promotes GLUT expression, regulates glycolysis, and enhances cell proliferation (Semenza, 2002). In a study investigating the feasibility of using auranofin (Figure 1B) to treat non-small cell lung cancer (NSCLC), tumor cell death was observed in Calu3 and HCC366 cells treated with 0.5 μm auranofin for 24–48 h, possibly due to the inhibition of the PI3K/Akt signaling pathway (Li et al., 2016). Auranofin may therefore be a potential candidate for the treatment of NSCLC.

The first step in glucose metabolism is glucose entry into the cell through GLUT embedded in the cell membrane. GLUT upregulation has been reported in many types of cancer and is believed to meet the large energy requirement of rapidly proliferating tumor cells (Macheda et al., 2005). Glucose metabolism in all cells must first go through glycolysis, which is occurs in the cytoplasm. In addition to GLUT, other glycolytic enzymes, such as hexokinase (HK), phosphofructokinase 1 (PFK1), and pyruvate kinase (PK), also play crucial roles in glucose metabolism. The expression levels of these enzymes affect tumor glycolysis, and, in turn, affect tumor proliferation; thus, these enzymes can be used as tumor markers (Hardt et al., 2000; Peng et al., 2008). A review has shown that inhibiting the tumor glycolysis pathway inhibits the proliferation of tumor cells and even kill tumor cells (Scatena et al., 2008).

PFK1 is the least efficient among the three rate-limiting enzymes and is therefore the most important regulatory point in the glycolytic pathway (Webb et al., 2015). Auranofin can inactivate PFK1 in human neutrophils, leading to ATP depletion in cells (Anderson et al., 1991). Cancer stem cells are capable of self-renewal and pluripotent differentiation, which are among the reasons for cancer metastasis and recurrence as well as resistance of tumor cells to chemotherapy and radiation therapy (Lobo et al., 2007). Hou et al. treated A549 and NCI-H460 cells with different concentrations of auranofin for 72 h, and their results showed that auranofin reduced the viability of tumor cells in a concentration-dependent manner, with half-maximal inhibitory concentration (IC_50_) values of 4 μM for A549 and 2 μM for NCI-H460 cells. Auranofin may inhibit glycolysis by inhibiting HK, thereby reducing glucose uptake and lactic acid production, leading to ATP depletion (Hou et al., 2018). Auranofin (4 μM) induced the depletion of cancer stem cells to 1.2% and impaired the tumorigenicity of cancer cells *in vivo* (Hou et al., 2018). In another anti-tumor study involving auranofin, researchers found that auranofin-treated human colon cancer cells HT29 had significantly reduced PK activity (Control, 18.4 ± 1.7 mU/ml; Auranofin, 12.6 ± 1.6 mU/ml) and lactic acid levels (Control, 100 ± 11.9%; Auranofin, 52.5 ± 1.1%) (Go et al., 2013). In the same study, auranofin was also found to oxidize a number of glycolytic enzymes, such as aldolase A (ALDOA), enolase 3 (ENO3), phosphoglycerate mutase 1 (PGAM1), LDHA, and PKM2. Tania Gamberi et al. studied the effects of the gold (III) compound Aubipyc (Figure 1C) on cisplatin-resistant A2780 ovarian cancer cells (2780/R) and cisplatin-sensitive A2780 ovarian cancer cells (2780/S) from the proteomic perspective. They found that A2780/S consumed less glucose after treatment with Aubipyc. They also observed decreased levels of glycolytic enzymes such as PKM, enolase 1 (ENO1), glyceraldehyde 3-phosphate dehydrogenase (GAPDH), and phosphoglycerate kinase 1 (PGK1) (Gamberi et al., 2014). They also studied the cytotoxicity of Aubipyc in cisplatin-resistant A2780 ovarian cancer cells. They found that this compound regulates glucose metabolism in tumor cells and significantly reduces lactate production through interaction with glycolytic enzymes such as aldolase C (ALDOC), LDHB, GAPDH, and PKM (Gamberi et al., 2015). This gold (III) compound could therefore downregulate glucose metabolism in tumor cells.

**FIGURE 2:**
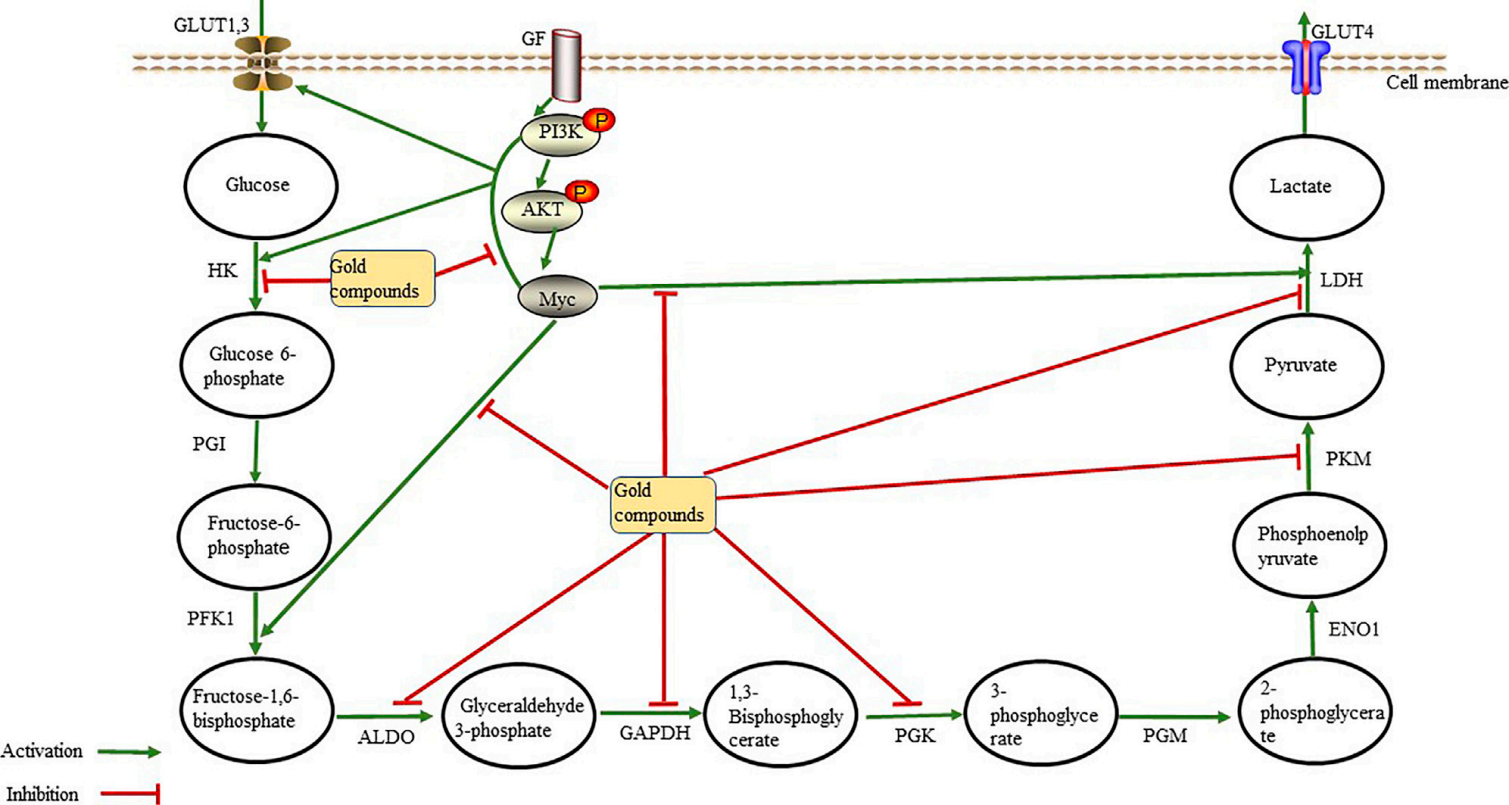
Gold compounds inhibit glycolytic enzymes through the phosphoinositide 3-kinase (PI3K)/Akt signaling pathway and the Myc oncogene. They also directly inhibit the activity of glycolytic enzymes, thereby reducing lactate and ATP production, leading to anti-tumor effects Abbreviations: ALDO, aldolase; ENO1, enolase; GAPDH, glceraldehyde-3-phosphate dehydrogenase; GF, growth factor; GLUT, gulcose transporter; HK, hexokinase; LDH, Lactate dehyrogenase; PFK1, phosphofructokinase 1; PGI, phosphoglucoisomerase; PGK, phosphoglycerate kinase; PGM, phosphoglcerate mutase; PKM, pyruvate kinase.

Normally differentiated cells use oxygen to “burn” glucose. The metabolism of glucose to lactic acid generates only two ATP molecules per glucose molecule, whereas complete oxidization of glucose molecule produces up to 36 ATP molecules. However, the production of ATP through glycolysis is faster than OXPHOS, which may be one of the reasons why tumor cells utilize glycolysis. Consequently, inhibition of glycolysis suppresses the growth of cancer cells. Thus, we hypothesize that gold compounds (Table 1) exert anti-tumor effects by interacting with glycolytic enzymes to reduce or inhibit ATP production during aerobic glycolysis (Figure 2).

**TABLE 1:** Effects of gold compounds on aerobic glycolysis metabolism in tumor cells.

Gold compounds	Cancer types	Subjects	Potential targets	Reference
[Au (d2pype)_2_] Cl	Human multiple myeloma	RPMI8226 cells	MYC ↓	Sze et al. (2020)
Auranofin	Human NSCLC	A549 and NCI-H460 cells and cancer stem cells	HK ↓	Hou et al. (2018)
	Human NSCLC	Calu3 and HCC366 cells	PI3K/Akt ↓	Li et al. (2016)
	Human colon cancer	HT29 cells	ALDOA↓,ENO3↓,LDHA↓, PGM1↓, and PKM2↓	Go et al. (2013)
Aubipyc	Human ovarian cancer	A2780/S	PKM↓,GAPDH↓,ENO1↓, and PGK1↓	Gamberi et al. (2014)
		A2780/R	ALDOC↓,LDHB↓,GAPDH↓ and PKM↓	Gamberi et al. (2015)

Abbreviations: ALDOA, aldolase A; ALDOC, aldolase C; ENO1, enolase 1; ENO3, enolase 3; HK, hexokinase; LDHA, lactate dehydrogenase A; LDHB, lactate dehydrogenase B; PGK1, phosphoglycerate kinase 1; PGM1, phosphoglycerate 1; PI3K, phosphoinositide 3-kinase; PKM2, pyruvate kinase M2; NSCLC, non-small cell lung cancer.

### Gold Compounds and Oxidative Phosphorylation

**FIGURE 3:**
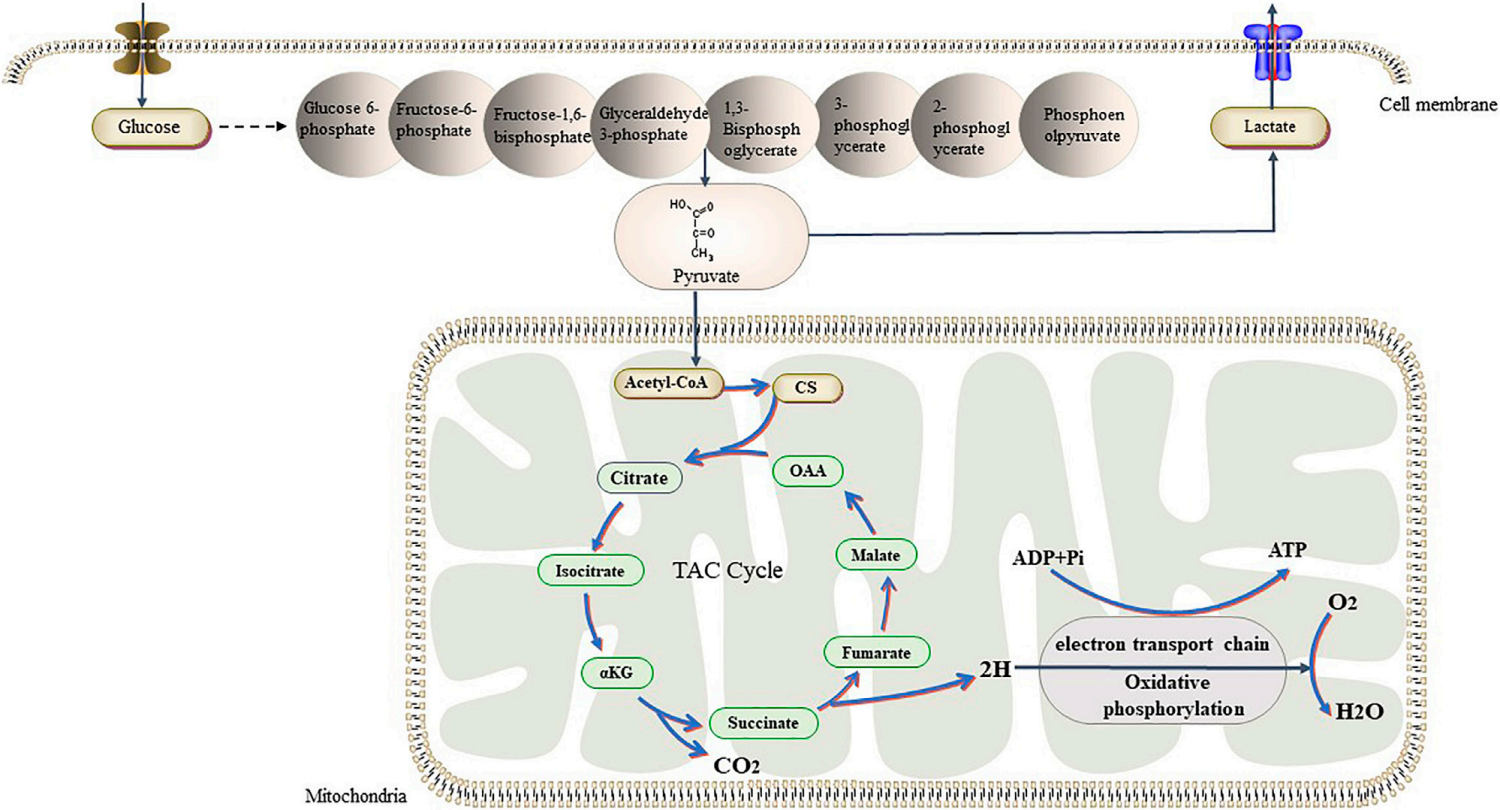
Tumor cells have different ways of glucose metabolism from normal cells. In the presence of sufficient oxygen, they also prefer glycolysis, while normal cells prefer aerobic glucose oxidation. Recent studies have shown that tumors can switch back and forth between aerobic glycolysis and oxidative phosphorylation in response to their own proliferative needs. Abbreviations: αKG, α-Ketoglutraic acid; Cd, Citrate synthase; OAA, Oxaloacetic acid.

Unlike tumor cells, normal cells mainly rely on the ATP produced through OXPHOS in the mitochondria for energy. Mitochondria contain more than 1,000 enzymes and proteins, which are distributed in the different parts of the mitochondria to metabolize glucose, fatty acids, proteins, etc. (Johannsen and Ravussin, 2009). The main function of the mitochondria is the conversion of stored chemical energy from nutrients into ATP through OXPHOS. For a long time, it has been believed that, in tumor cells, glycolysis is enhanced and OXPHOS is downregulated due to mitochondrial dysfunction. However, recent studies have shown that OXPHOS is upregulated in Hodgkin lymphoma, pancreatic ductal adenocarcinoma, breast cancer, and leukemia, and that these tumors rely on OXPHOS to obtain ATP (Whitaker-Menezes et al., 2011; Viale et al., 2014; Birkenmeier et al., 2016). In 2019, Duan et al. showed that low environmental glucose levels induced higher oxygen consumption in HCT116 cells, and that tumor cells may switch back and forth between glycolysis and OXPHOS in response to metabolic challenges and changes in the TME (Elgendy et al., 2019) (Figure 3). Another study showed that lactic acid can induce localized energy metabolic reprogramming, which is key to regulating tumor glycolysis and oxidative phosphorylation in glioblastoma (Duan et al., 2018). In case of lactic acidosis (20 mM lactic acid, pH 6.7), tumor cells were found to rely primarily on OXPHOS (86.3–94.3%) rather than on glycolysis (5.3–13.4%) for ATP production (Wu et al., 2016). Some researchers have found the tumor model of *Drosophila melanogaster* had increased efficiency of mitochondrial membrane fusion-mediated OXPHOS and increased rates of NADH/NAD + metabolism increased. The neural stem cell tumors in the tumor model also had high oxidation, which promotes tumor immortality; however, the underlying mechanisms remain unclear (Bonnay et al., 2020).

The respiratory chain is a reaction system composed of a series of redox complexes (hydrogen transmitters and electron transmitters) embedded in the inner membrane of the mitochondria (Dudkina et al., 2005). Chiara et al. found that the gold (III)-dithiocarbamate compound AUL12 (Figure 1P) induced oxidative stress and tumor cell death by inhibiting the respiratory chain complex I and releasing reactive oxygen species (ROS). This then activated glycogen synthase kinase 3 (GSK-3α/β) and the Bcl-2-associated X protein (Bax), leading to the opening of the mitochondrial permeability transition pore opening and to tumor cell death (Chiara et al., 2012). Usually, 90% of the molecular oxygen in the human body is consumed through the mitochondrial respiratory chain system, and the mitochondria are the primary sites of ROS production in eukaryotic cells (Dan Dunn et al., 2015). If ROS is not effectively cleared, the excessive levels of ROS can damage mitochondrial DNA (mtDNA) due to the lack of histone protection and the lack of an effective repair system. ROS is a double-edged sword. The accumulation of ROS above the death threshold can lead to apoptosis, including that of tumor cells (Martin and Barrett, 2002). However, the accumulation of mtDNA damage is closely related to tumors (Durham et al., 2003), and a study has shown that mtDNA damage results in tumor invasion in breast cancer (Yuzefovych et al., 2016). Therefore, the regulation of ROS is crucial for cancer treatment.

In 1999, researchers discovered that aurothioglucose (ATG), a gold (I) compound, enhances coxsackievirus virulence by inhibiting thioredoxin (Trx) activity and by increasing oxidative stress levels in mice (Smith et al., 1999). Oxidative stress, which is caused by increased mitochondrial dysfunction, glucose deprivation, and elevated levels of nicotinamide adenine dinucleotide phosphate (NADPH), is a pathological feature of tumors (Spitz et al., 2000; Cook et al., 2004; Reinehr et al., 2007). To maintain survival under high ROS levels, tumor cells have developed a set of strong antioxidative defense mechanisms, including changes in the activity and levels of members of the Trx system, such as NADPH, Trx, and thioredoxin reductase (TrxR) (Arner and Holmgren, 2000). An important mechanism of a number of anti-tumor approaches, such as radiotherapy and several chemotherapeutics, is the selective killing of cancer cells by inducing high levels of ROS. Thus, weakening the oxidative adaptation of tumors will improve the efficacy of anti-tumor therapy.

In 1967, researchers discovered the presence of Trx in rat tumors (Moore, 1967). Superoxide anions produced *via* mitochondrial aerobic respiration stimulate the production of large amounts of hydrogen peroxide catalyzed by superoxide dismutase; TrxR eliminates ROS to protect cells from oxidative stress (Holmgren and Lu, 2010). TrxR has been a target of anti-tumor therapy in mammals. Arsenic trioxide (ATO), a TrxR inhibitor that induces tumor cell apoptosis primarily by enhancing the oxidative stress response, has been approved for the treatment of acute promyelocytic leukemia (Lu et al., 2007). Liu et al. found that gold (III) Schiff-base compounds (Figure 1D) inhibited the activity of TrxR, thereby elevating ROS, mediating endoplasmic reticulum stress, and leading to mitochondrial dysfunction, which then inhibited the growth of hepatocellular carcinoma (Bian et al., 2020b). They also confirmed that a gold (I) compound containing an oleanolic acid derivative (Figure 1E) had a potential anti-ovarian cancer effect by inhibiting TrxR and activating ROS-mediated endoplasmic reticulum stress (Bian et al., 2020a).

Thus, breaking the REDOX balance in the tumor environment seems to be an effective strategy for cancer treatment. Phosphine gold (I) compounds inhibit the activity of TrxR in the cytoplasm and mitochondria, leading to an accumulation of intracellular ROS, which induces cytotoxicity in tumor cells. These compounds also activate caspase-3 to induce cell death in A549, HCT-15, and HeLa cells. Phosphine gold (I) compounds were also found to inhibit the activity of glutathione reductase and peroxidase in human ovarian cancer cells (Gandin et al., 2010). Mitochondria consume ROS in a substrate-dependent and respiration-dependent manner, predominantly *via* the thioredoxin/peroxiredoxin (Trx/Prx) system (Lopert et al., 2012). Three gold (I) compounds, auranofin, chloro (triethylphosphine) (TEPAU), and aurothiomalate, could inhibit the activity of TrxR and stimulate the permeability transition and release of cytochrome c in rat liver mitochondria (Rigobello et al., 2004). Auranofin inhibits TrxR activity and OXPHOS in HCT 116 cells, leading to mitochondrial dysfunction and tumor cell death. An artemisinin-derivative-(NHC) gold (I) compound could inhibit nuclear factor erythroid 2–related factor 2 (NRF2) transcriptional activity in tumor cell models, including those of solid tumors (prostate, bladder, bone, lung, breast, and liver) and hematological tumors (chronic myelogenous leukemia and acute myeloid leukemia) (Zhang et al., 2020). High concentrations of auranofin inhibit proteasome activity, which may be related to auranofin’s inhibitory effects on cysteine deubiquitinating enzyme (DUB) USP14 (Zhang et al., 2019). Gold (III) compound benzyl bis(4-cyclohexyl-3-thiosemicarbazonate) (Figure 1F) inhibits intracellular TrxR activity, resulting in an imbalance in the cellular redox status, thereby inducing MCF7 cell death (Rodriguez-Fanjul et al., 2018). Rouco et al. also found that two gold (I) compounds, Au(pben)(PPh_3_) and Au(pben)(PEt_3_) (Figure 1H), can induce apoptosis of SH-SY5Y neuroblastoma cells through oxidative stress. The mechanism of action of these compounds is related to the inhibition of TrxR, and the resulting ROS affects mitochondrial polarization and induces caspase-3 production (Rouco et al., 2020). Benzimidazol-2-ylidene gold (I) compounds (Figure 1G) act on TrxR and inhibit mitochondrial respiration, leading to intracellular accumulation of ROS and the induction of apoptosis in cancer cells (Rubbiani et al., 2010). When MDA-MB-231, HT-29, and vincristine-resistant NALM-6 leukemia cells were exposed to gold (I) compounds with phosphane and thiotetrazolate ligands (Figure 1I), investigators found that cell proliferation was inhibited, and that cytotoxicity was attributed to the strong inhibition of TrxR by the compound (Serebryanskaya et al., 2015). Gold (I) NHC compound MC3 (Figure 1K) efficiently inhibits the proliferation of gemcitabine-resistant pancreatic cancer cells by inhibiting TrxR activity. These compounds could inhibit the activity of TrxR, keep Trx in the oxidized state, release free apoptosis signal-regulating kinase 1 (ASK1), and, in turn, activate the p38-MAPK pathway to promote tumor cell apoptosis (Cheng et al., 2014). A proteomic study (Magherini et al., 2018) on the effect of Gold (I)-N-heterocyclic carbene compounds on tumor cell proliferation showed that Au(NHC) and Au(NHC)_2_ (Figure 1S), both of them can inhibit glucose uptake and reduce oxygen consumption of A2780 cells, the latter can also inhibit mitochondrial respiration coupled with a decrease of citrate synthase (CS) amount, the rate-limiting enzyme of TCA cycle. Au(NHC)_2_ treatment also leads to strong antiproliferative effects of A2780 cells, potent inhibition of TrxR activity, decrease of mitochondrial respiration coupled with a lower mitochondrial membrane potential and higher glycolytic activity followed by a decrease of ATP level. An NMR metabolomics study (Ghini et al., 2021)on the effects of auranofin on A2780 cells showed that within the first 12 h of A2780cells treated by auranofin, the TCA cycle activity of tumor cells was inhibited and glycolysis was upregulated. Of greatest concern are the increased concentrations of glutathione in tumor cells and the upregulation of proteins involved in glutathione synthesis. (Saei et al., 2020) also found that auranofin interfered with glutathione metabolism and oxidative stress responses in tumor cells (HCT116, A375, RKO). TrxR, cysteine and histidine-rich domain containing protein 1 (CHORDC1) and NFkB2 are the targets of auranofin’s chemical activity.

He et al. demonstrated that cancer-targeted mesoporous silica nanoparticles (MSNs) for the delivery of a gold (III) porphyrin complex [Au-1a@MSN(R)] (Figure 1L) can suppress the TrxR system, which may subsequently trigger ROS-mediated apoptosis signaling, such as ERK and AKT signaling, in A549 cells (He et al., 2014). Encapsulation of gold (I) compounds [Au(I)] into hydrophobic domains of nanoparticles (NPs), resulting in Au(I)-loaded NPs (Au(I)⊂NPs) (Figure 1M), led to constructs that had the ability to kill cancer cells. Gold (I) can inhibit TrxR in MCF-7 human breast cancer cells and can lead to intracellular ROS accumulation, thereby inducing autophagy and apoptosis. Au(I)⊂NPs blocks autophagy, leading to excessive depletion of organelles and essential proteins, ultimately resulting in cell death (Lin et al., 2015). Vela et al. found that three different iminophosphorane-organogold (III) compounds (compound 1, a neutral compound with two chloride ligands; compound 2, a cationic compound with a dithiocarbamate ligand; and compound 3, a cationic compound with a water-soluble phosphine and a chloride ligand) (Figure 1O) induced mitochondrial depolarization and apoptosis or necrosis of leukemia cells. The mechanism by which gold compounds induce apoptosis or death of leukemia cells is related to ROS. Necrosis induced by compounds 1 and 2 was Bax/(Bcl-2-agonist killer (Bak)- and caspase-independent, while apoptosis induced by compound 3 was Bax/Bak-dependent (Vela et al., 2011). Meanwhile, bimetallic titanium-gold-containing compounds Titanocref and Titanofin (Figure 1J) can significantly inhibit angiogenesis and growth of xenografted clear cell renal cell carcinoma (cCRCC) Cak-1 tumors in NOD. CB17-PRKDC SCID/J mice. In this case, the mechanism of action of the gold compounds is related to the ROS-mediated c-Jun N-terminal kinase/mitogen-activated protein kinase (JNK/MAPK) apoptosis signaling pathway (Elie et al., 2020).

**FIGURE 4:**
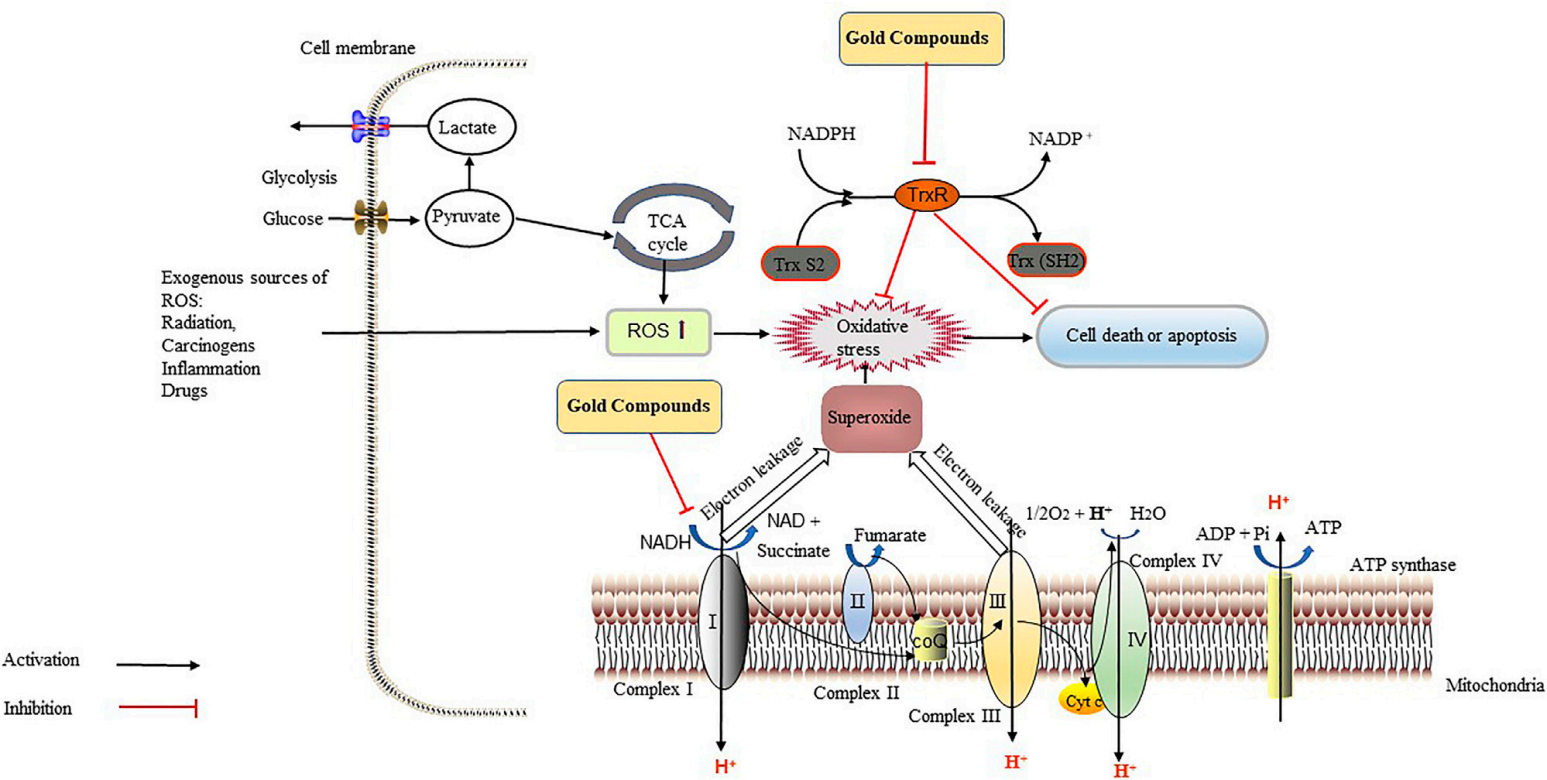
Gold compounds exert anti-tumor effects through the inhibition of thioredoxin reductase (TrxR) and the respiratory chain. TrxR maintains the redox balance in tumor cells, reverses the damage caused by various types of oxidatives stress, accelerates cell division and growth, and increases the ability of tumor cells to resists apoptosis, which all accelerate the metastasis and diffusion of cancer cells. Gold compounds can inhibit the activity of TrxR, leading to the accumulation of reactive oxygen species (ROS) and oxidative stress in tumor cells, thereby causing tumor cells death. Gold compounds also inhibit the respiratory chain complex I, causing intracellular ATP depletion, which inhibits tumor growth. Abbreviations: CoQ, coenzyme Q; Cyt C, cytochrome C; NAD, nicotinamide adenine dinucleotide; NADPH, NAD phosphate.

Bis-[1,2-bis(diphenylphosphino)-ethane]gold (l) lactate {[Au (dppe)2]+} can selectively damage mitochondrial function in rat hepatocytes, and the mechanism is related to the uncoupling of OXPHOS, which induces cell death (Smith et al., 1989). The compound causes oxidation and phosphorylation uncoupling, such that oxidation can still proceed, while phosphorylation cannot. The compound increased the permeability of the mitochondrial intima to H+, thereby eliminating the transmembrane gradient of H+; as a result, ATP is not generated, and cellular activity is inhibited (Kadenbach, 2003). Normal mitochondrial membrane potential (MMP) is a prerequisite for maintaining mitochondrial OXPHOS and ATP production. When an etiology leads to the disturbance of the electron transport process in the respiratory chain and affects the formation of the H+ transmembrane gradient, the MMP drops (i.e., the mitochondrial membrane is depolarized). Jellicoe et al. also found that a new lipophilic, cationic gold (I) N-heterocyclic carbene compound (Figure 1N **)** could significantly inhibit the growth of tumorigenic liver progenitor cells (PIL2). The gold compound depolarized the MMP, depleted ATP, and activated caspase-3 and caspase-9, suggesting that apoptosis was mediated by mitochondrial processes (Jellicoe et al., 2008). Therefore, gold compounds (Table 2 **)** can be used for cancer treatment by targeting TrxR, regulating oxidation reactions, and acting on the mitochondria, which all lead to tumor cell apoptosis (Figure 4).

**TABLE 2:** Gold compounds inhibit the growth of tumor cells by acting on ROS through various targets.

Gold compounds	Cancer types	Subjects	Potential targets	Reference
Gold(III) Schiff-base compounds	Human hepatocellular carcinoma	HepG2 cells	TrxR↓ and ROS↑	Bian et al. (2020b)
Gold(I) compound containing an oleanolic acid derivative	Human ovarian cancer	A2780 cells	TrxR↓ and ROS↑	Bian et al. (2020a)
Phosphine Gold(I) Compounds	Human NSCLC, colon cancer, cervical cancer	A549, HCT-15, and HeLa cells	TrxR↓, glutathione reductase↓, peroxidase↓, and caspase-3↑	Gandin et al. (2010)
Auranofin	Human colon cancer	HCT 116 cells	TrxR↓, OXPHOS↓, and proteasome ↓	Zhang et al. (2019)
Artemisinin-derivative-(NHC) gold(I) compound	Human solid tumors (prostate, bladder, bone, lung, breast, liver) and human hematological tumors (CML, AML)	Tumor cell models including solid tumors (prostate, bladder, bone, lung, breast, liver) and human hematological tumors (CML, AML)	NRF2↓ and ROS↑	Zhang et al. (2020)
Gold (III) compound benzil bis	Human breast cancer	MCF7 cells	TrxR↓	Rodriguez-Fanjul et al. (2018)
Au (pben) (PPh_3_) and Au (pben) (PEt_3_)	Human neuroblastoma	SH-SY5Y neuroblastoma Cells	TrxR↓and ROS-autophagy↑	Rouco et al. (2020)
Benzimidazol-2-ylidene gold(I) compounds	Human liver cancer, human breast cancer, human colon cancer	HEP-G2, MCF-7, HT29 and HCT-116	TrxR↓ and mitochondrial respiration↓	Rubbiani et al. (2010)
Gold(I) compounds with phosphane and thiotetrazolate ligands	Human breast cancer, human colon cancer, leukemia	MDA - MB - 231, HT-29 and vincristine resistant NALM-6 leukemia cells	TrxR↓	Serebryanskaya et al. (2015)
Bimetallic titanium-gold-containing compounds	Human cCRCC	NOD.CB17-PRKDC SCID/J mice bearing Xenograft cCRCC cak-1 tumors	ROS-JNK/MAPK↑	Elie et al. (2020)
Gold(I) NHC compounds MC3	Human pancreatic cancer	Pancreatic cancer cells	TrxR↓ and ASK1-p38-MAPK signaling↑	Cheng et al. (2014)
Au(NHC) and Au(NHC)_2_	Human ovarian cancer	A2780 cells	TrxR↓, mitochondrial respiration↓	Magherini et al. (2018)
Auranofin	Human ovarian cancer	A2780 cells	TCA cycle↓, glutathione↑	Ghini et al. (2021)
Auranofin	Human colorectal carcinoma cells Human skin malignant melanoma cells Human colon carcinoma cells	HCT116 A375 RKO	TrxR↓, CHORDC1↑,NFkB2↑	Saei et al. (2020)
Au-1a@MSN(R) NPs	Human NSCLC	A549 cells	TrxR↓, ROS-ERK↑, and AKT signaling↑	He et al. (2014)
Au(I)-loaded NPs (Au(I)⊂NPs)	Human breast cancer	MCF-7 cells	TrxR↓and ROS-autophagy↑	Lin et al. (2015)
Gold (III)-dithiocarbamate compound AUL12	Human osteosarcoma	SAOS-2 cells	Respiratory chain↓ and ROS↑	Chiara et al. (2012)
Lipophilic, cationic gold(I) N-heterocyclic carbene compound	Human tumorigenic liver cancer	Tumorigenic liver progenitor cell (PIL2)	Mitochondria↑, caspase-3↑ and caspase-9↑	Jellicoe et al. (2008)
Iminophosphorane-organogold (III) compounds	Human leukemia	T-cell leukemia Jurkat (clone E6.1)	Mitochondria↑ and ROS-Bax/Bak↑	Vela et al. (2011)

Abbreviations: ASK1, apoptosis signal-regulating kinase 1; Bak, Bcl-2-homologous antagonist/killer; Bax, Bcl-2-associated X protein; cCRCC, clear cell renal carcinoma; ERK, extracellular signal-regulated kinase; JNK, c-Jun N-terminal kinase; MAPK, mitogen-activated protein kinase; NPs, nanoparticles; NRF2, nuclear factor erythroid 2-related factor 2; NSCLC, non-small cell lung cancer; OXPHOS, oxidative phosphorylation; ROS, reactive oxygen species; TrxR, thioredoxin reductase.

## The Effects of Gold Compounds on Protein and Nucleic Acid Metabolism in Tumors

### Gold Compounds and Protein Metabolism

Gold compounds not only affect glucose metabolism, but also affect protein and nucleic acid metabolism in tumor cells. In normal tissues, protein synthesis and degradation are in dynamic equilibrium, and some of the synthesized proteins are rapidly degraded by proteasomes (Schubert et al., 2000). In the process of protein metabolism, misfolding often occurs and is closely linked to cancer (Scott and Frydman, 2003). Two main pathways for protein degradation are utilized by mammalian cells: the ubiquitin-proteasome pathway (UPS) and the autophagy-lysosome pathway (ALP). The 26S proteasome is a proteolytic complex consisting of the 20S proteasome and the 19S regulatory particle that facilitate the degradation of ubiquitinated substrates (Voges et al., 1999). Actively proliferating malignant cells have been shown to be more sensitive to proteasome inhibitors than normal cells, and proteasome inhibitors can increase the sensitivity of tumors to chemotherapy (Ma et al., 2003). Gold-dithiocarbamate compounds, gold (I) [Au (ESDT)]_2_, and gold (III) [AuBr_2_(ESDT)] (Figure 1P), can inhibit 20S and 26S proteasome activity in human breast cancer MDA-MB-231 cells, resulting in the accumulation of ubiquitinated proteins and proteasome target proteins, and inducing the death of tumor cells (Zhang et al., 2010). Micale et al. confirmed that six gold compounds {gold (III) compounds: K [Au(Sac)_3_Cl], (pbi)Au(OAc)_2_, Aubipyc, Auoxo6, and Au_2_phen; and a gold(I) compound: [(pbiH)Au(PPh_3_)]PF6} inhibited 20S proteasome activity, providing evidence for the potential of proteasome-targeted gold compounds as anticancer drugs (Micale et al., 2014). Auranofin inhibited the proliferation of LNCaP and 22RV1 prostate cells (PCa) and suppressed the growth of subcutaneous xenografts of PCa in nude mice, which was associated with inhibition of the 19S proteasome (Liu et al., 2019). Cathepsins are cysteine proteases found in various animal tissues, specifically in lysosomes, and have been found to be involved in the regulation of apoptosis (Chwieralski et al., 2006). An increasing number of studies have shown that lysosomes are closely related to the occurrence of cancer. For example, cathepsin D is an important factor in the recurrence and death of breast cancer; cathepsin B is related to the development of tumors from a precancerous to a malignant state; and cathepsin L expressed in tumors may bind nuclear transcription factors, which may affect cell proliferation (Sloane, 1990; Tandon et al., 1990; Sullivan et al., 2009). Zhu et al. demonstrated that cycloaurated gold (III) compounds (Figure 1Q) inhibit the activity of cathepsins B and K in the HT29 human colon tumor xenograft model (Zhu et al., 2011). Thus, enzymes involved in protein degradation play important roles in cancer progression. Gold compounds targeting protein metabolic enzymes (Table 3) have high specificity and may be precisely regulated, making them useful tools for cancer treatment.

**TABLE 3:** Gold compounds inhibit tumor cell growth by acting on proteasomes or by inhibiting nucleic acid synthesis.

Gold compounds	Cancer types	Subjects	Potential targets	Reference
Gold (I) [Au (ESDT)]_2_ and gold(III) [AuBr_2_(ESDT)]	Human breast cancer	MDA-MB-231 cells	20S and 26S Proteasome ↓	Zhang et al. (2010)
Auranofin	Human prostate cancer	Lncap and 22RV1 cells	19S Proteasome↓	Liu et al. (2019)
Cycloaurated gold (III) compounds	Human colon cancer	HT29 cells	Proteases B and K↓	Zhu et al. (2011)
Ph_3_PAuIm (CN)_2_ Ph_3_PAuIm (Cl)_2_	Human breast cancer	SKBR3 and A17 cells	DHFR↓ and TrxR↓	Galassi et al. (2020)

Abbreviations: DHFR, dihydrofolate reductase; TrxR, thioredoxin reductase.

### Gold Compounds and Nucleic Acid Metabolism

Nucleic acids are basic components of all known forms of life. It plays an important role in biological metabolism and is closely correlated with cell growth and division. DNA is believed to play an important role in cell division, whereas RNA mainly guides protein synthesis. In tumor cells, nucleic acid decomposition is significantly reduced, and DNA and RNA content is increased to allow the rapid proliferation of tumor cells. Dihydrofolate reductase (DHFR) is involved in the reproduction and replication of cancer cells in humans. Its main function is to reduce dihydrofolate to tetrahydrofolate, and then synthesize tetrahydrofolate coenzymes, which participate in the synthesis of nucleic acids and amino acids and promote the growth of cancer cells (Askari and Krajinovic, 2010). Two compounds [4,5-dicyano-imidazolate-1-yl-gold (I)-triphenylphosphane Ph_3_PAuIm (CN)_2_ and 4,5-dichloro-imidazolate-1-yl-gold (I)-triphenylphosphane Ph_3_PAuIm (Cl)_2_] (Figure 1R) significantly inhibited the activity of DHFR and TrxR in SKBR3 and A17 cells (Galassi et al., 2020). DHFR inhibitors selectively bind DHFR, which prevents the conversion of dihydrofolate to tetrahydrofolate, blocks folic acid metabolism, and interferes with DNA and protein synthesis, eventually leading to cell death. Thus, DHFR is considered an important target for the development of antitumor drugs.

## Conclusion

Since the discovery of the anti-tumor activity of auranofin, an increasing number of ligand-bound gold compounds have been used in anticancer research. Gold (I) compounds used in biological research mainly include gold (I) phosphine compounds and organometallic gold (I) compounds, whereas gold (III) compounds mainly include gold (III) compounds with tetradentate, tridentate, and tidentate ligands (Casini et al., 2018). Here, we have discussed that gold compounds exhibit anti-tumor effects primarily by targeting metabolic pathways or metabolic products of tumor cells. We have also presented the efficacy of gold compounds for the treatment of cancer in *in vitro* and *in vivo* models. The gold compounds that exhibit the potential as anticancer agents include: auranofin, which inhibits HK and PFK and disrupts the REDOX balance in tumors; gold-dithiocarbamate compounds, which inhibit proteasome activity and RNA synthesis; and binuclear gold (III) compounds, which inhibit cathepsin activity.

Although increased aerobic glycolysis has been widely used as a metabolic marker for cancer cells, most cancer cells still have mitochondrial function, suggesting that glycolysis is not the only way tumor cells produce energy. In addition, the ATP produced by the oxidative phosphorylation of tumor cells was basically the same as that of normal cells, and the glucose uptake of tumor cells was much higher than that of normal cells. As mentioned in the first part of the article, gold compound can directly or indirectly inhibit the glycolytic enzyme activity of tumor cells and reduce the production of ATP, so as to achieve the effect of anti-tumor proliferation. However, in another study of the anti-proliferation of tumor cells by gold compounds, the glycolytic activity of A2780 cells treated with Au(NHC)_2_ was upregulated. Au(NHC)_2_ can reduce mitochondrial respiration, alter mitochondrial membrane potential, damage the mitochondrial function, and induce the apoptosis of tumor cells (Magherini et al., 2018). The enhanced glycolysis activity, decreased oxygen consumption, and increased lactic acid production reflected the damage of gold compounds to the respiratory function of tumor cells, which may be the embodiment of the metabolic compensatory mechanism of tumor cells in response to the cytotoxicity caused by gold compounds.

Unlike platinum-based metal compounds, which target nucleic acids, gold compounds seem to prefer to bind to proteins. Auranofin has been shown to have a special binding preference for proteins containing free cysteine and free selenocysteine. Free cysteine residues are the main binding sites of gold compounds, which exert cytotoxic effects by directly blocking the active sites cysteine or selenocysteine of proteins (Zoppi et al., 2020). TrxR is indeed the target of many gold compounds for their anti-tumor proliferative properties and it has a C-terminal active site motif, Gly-Cys-Sec-Gly. Both the cysteine and selenocysteine residues play an irreplaceable role in maintaining TrxR’s physiological properties. As mentioned above, Trx and GSH redox systems work together to maintain intracellular redox stability. Gold compounds target TrxR and disrupt REDOX homeostasis in tumor cells. This is the reason why glutathione activity is activated in A2780 cells treated with auranofin, which is the feedback mechanism of tumor cells in response to auranofin damage (Ghini et al., 2021). We hypothesized that, in addition to directly inhibiting the ATP needed for tumor cell proliferation, gold compounds also act on a variety of metabolic pathways to induce ROS accumulation, which is the main mechanism through which gold compounds induce tumor cell apoptosis.

Evidently, tumor cells have high metabolic adaptability, and they automatically switch over or activate other pathways when they encounter stress injuries. As such, we believe that cancer is a metabolic disease. We propose that tumor nutrition and metabolic regulation should become main targets of tumor therapy. Further understanding of the metabolic changes would allow us to identify the alternative metabolic targets for the development and selection of effective anticancer drugs. As we have discussed, the glycolytic pathway is not the only metabolic target for cancer treatment, and we should adjust treatment strategies accordingly. The ideal therapeutic strategy would involve the regulation of tumor metabolism though the blockade and regulation of multiple metabolic pathways. As we have presented here, gold compounds target several metabolic pathways in tumor cells and induce changes to the tumor microenvironment. Thus, the use of gold compounds is a promising strategy for cancer therapy.
